# FFPred 2.0: Improved Homology-Independent Prediction of Gene Ontology Terms for Eukaryotic Protein Sequences

**DOI:** 10.1371/journal.pone.0063754

**Published:** 2013-05-22

**Authors:** Federico Minneci, Damiano Piovesan, Domenico Cozzetto, David T. Jones

**Affiliations:** 1 Bioinformatics Group, Department of Computer Science, University College London, London, United Kingdom; 2 Biocomputing Group, Department of Biology, University of Bologna, Bologna, Italy; University of Alberta, Canada

## Abstract

To understand fully cell behaviour, biologists are making progress towards cataloguing the functional elements in the human genome and characterising their roles across a variety of tissues and conditions. Yet, functional information – either experimentally validated or computationally inferred by similarity – remains completely missing for approximately 30% of human proteins. FFPred was initially developed to bridge this gap by targeting sequences with distant or no homologues of known function and by exploiting clear patterns of intrinsic disorder associated with particular molecular activities and biological processes. Here, we present an updated and improved version, which builds on larger datasets of protein sequences and annotations, and uses updated component feature predictors as well as revised training procedures. FFPred 2.0 includes support vector regression models for the prediction of 442 Gene Ontology (GO) terms, which largely expand the coverage of the ontology and of the biological process category in particular. The GO term list mainly revolves around macromolecular interactions and their role in regulatory, signalling, developmental and metabolic processes. Benchmarking experiments on newly annotated proteins show that FFPred 2.0 provides more accurate functional assignments than its predecessor and the ProtFun server do; also, its assignments can complement information obtained using BLAST-based transfer of annotations, improving especially prediction in the biological process category. Furthermore, FFPred 2.0 can be used to annotate proteins belonging to several eukaryotic organisms with a limited decrease in prediction quality. We illustrate all these points through the use of both precision-recall plots and of the COGIC scores, which we recently proposed as an alternative numerical evaluation measure of function prediction accuracy.

## Introduction

The picture of human biology is becoming more and more complex, given the discovery of novel functional elements and the observations of widespread alternative splicing events [1], [2], [3]. Currently, UniProtKB [4] includes more than 131,000 human protein chain entries, 8% of which have received Gene Ontology (GO) [5] annotations based on functional assays. However, 30% of these entries carry no functional information at all – a figure unlikely to change, even if we were to consider data waiting for inclusion into public databases. The combinatorial complexity of gene products, biological functions and cellular conditions makes systematic *in vitro* or *in vivo* testing impracticable; furthermore, proteins are not equally amenable to different experimental protocols.

Computational methods can be useful, but their reliability rests on our limited understanding of how function diverges as sequence, structure or environmental conditions vary. The most popular tools detect conserved patterns of sequence or structural features, ranging from short motifs to domains and domain arrangements [6]. Most sequence-similarity based approaches produce fairly detailed annotation transfers, when evolutionary relationships to previously characterized proteins can be confidently established. Structure-based techniques build on the assumption that structural conservation reflects better protein functional similarity, but their use is constrained by the data available and by the difficulty of assessing the statistical significance of structural matches.

A few attempts have been made to make functional assignments for proteins with distant or no detectable homologues with experimentally characterized function [7]. The ProtFun method pioneered the idea of transferring functional annotations between human proteins with similar biophysical attributes – e.g. the occurrence of charged amino acids, low complexity regions, signal peptides and trans-membrane helices and post-translationally modified residues [8], [9]. The possibility of identifying such features with sufficient accuracy and of linking them explicitly to particular biological roles is key to the viability of this approach. The current running server uses artificial neural networks to integrate the output of independent feature predictors, and consequently annotates protein sequences with cellular roles, enzyme classes and selected GO categories for which robust statistical models could be learned. FFPred took this approach further by considering the strong correlation between the length and position of intrinsically disordered protein regions with some molecular activities and biological processes [10], [11]. Similar to ProtFun, FFPred was initially conceived for orphan human proteins, but it generalizes well to other vertebrate proteomes too.

Programs for data integration have also emerged, which leverage on genome-wide datasets including protein interactions with other proteins or nucleic acids, as well as metabolic, signalling and gene regulatory networks. These hold the promise of increasing prediction scope and accuracy, but their usefulness is still hampered by the noisy and heterogeneous nature and by the limited taxonomic coverage of the underlying measurements and data [12].

Here, we present an updated version of FFPred, which results from a different training procedure coupled to larger protein sequence and annotation databases. We first describe how the list of predicted GO term has changed and then provide evidence for improved performance. We confirm that prediction accuracy is acceptable also for other model organisms’ proteins, supporting the use of FFPred 2.0 for eukaryotic function prediction. Finally, we describe the integration of the new tool within the resources maintained by the UCL Bioinformatics Group at http://bioinf.cs.ucl.ac.uk/psipred/.

## Materials and Methods

### Training of FFPred 2.0

Selection of proteins and GO terms. Annotations for human proteins were obtained from the Gene Ontology Annotation (GOA) [13] database, version 26 June 2012, while amino acid sequences were retrieved from UniProtKB [4] version 2012_06. The Gene Ontology (GO) OBO file version 1.3281 was used for term definitions and semantic relations [5]. Only GO terms belonging to the molecular function (MF) or biological process (BP) domains were considered. An initial set of 39,971 proteins with at least one such functional annotation and maximum sequence length of 1,500 amino acids was initially selected; 22,528 representatives with less than 90% pair-wise sequence identity were subsequently identified with CD-HIT [14].

A support vector regression model was learnt for a term *t* if:

- *t* also occurs in UniProtKB/SwissProt;
- there are at least 150 proteins (positive examples) annotated in GOA with *t* or its descendants;
- there are at least 500 proteins (negative examples) that are (i) not labelled with *t*, its descendants and its ancestors; and (ii) nonetheless bear at least 2 MF terms and 2 BP terms with evidence code other than IC, NAS, TAS and IEA.

Both “is a” and “part of” relations were followed in the graph to collect ancestor and descendant terms.

Protein features. FFPred 2.0 employs the same feature encoding scheme as described before [10]. Table 1 lists the software currently used for the prediction of sequence-based features, which are still grouped into 14 sets – see table S2 in [10]. Due to changes in WoLF-PSORT [15] and SignalP [16] output, the corresponding feature lists were updated.

**Table 1:** Software for feature prediction.

Feature Group	Program	Reference
Amino acid composition	in-house C++ code	NA
Sequence features	in-house C++ code	NA
Transmembrane segments	MEMSAT-SVM	[27]
Secondary structure	PSIPRED 3.3	[28]
Intrinsically disordered regions	DISOPRED 2.43	[29]
Signal peptides	SignalP 4.0	[16]
Subcellular localization	WoLF PSORT 0.2	[15]
PEST regions	epestfind in EMBOSS 6.4.0	[30]
Low complexity regions	Pfilt	[31]
Coiled coils	COILS 2.2	[32]
Phosphorylation sites	NetPhos 3.1	[33]
N-linked glycosylation sites	NetNGlyc 1.0c	[33]
O-GalNAc-glycosylation sites	NetOGlyc 3.1d	[34]

For any input amino acid sequence, FFPred 2.0 calculates several feature groups that are listed in the first column. The second and third columns report the name of the package used and the relevant literature citation respectively.

Preparation of the protein sets. All positive and negative sets were partitioned for later use in the k-fold cross validation and testing procedures. For every set, 30% of the proteins were randomly selected and kept aside for final validation. The remaining proteins were partitioned into 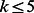 equally sized groups with at least 35 proteins; also, positive and negative sets for the same GO term needed to have the same number of partitions. The highest value of k that satisfied these criteria was used. The groups were created using a custom-made algorithm that ensured equal group size, while clustering proteins according to their relative distances as in standard K-medoids clustering algorithms [17]. For each pair of proteins, the distance used was a measure of the homology relationship between them, calculated as the minimum of all of the alignment E-values obtained using bl2seq (from the BLAST 2.2.26 package [18]) on the two protein sequences.

Training of the SVMs. The SVM-Light package version 6.02 [19] was used to train one binary classifier for each of the GO terms. For a given GO term, the procedure used was as follows.

Firstly, a series of cross validation steps was performed that ensured optimisation of kernel type, kernel parameters, and list of useful features at the same time. The kernels used were linear and radial basis function (RBF) kernels. Optimised kernel parameters were C in the linear case, C and γ in the RBF case. Features in each of the 14 different groups, described in section “Protein features” above, were either all included or the whole group discarded. The cross validation procedure tested all combinations of kernel type and kernel parameters using an exhaustive grid search, initially using all feature groups in the input files, and then iteratively removing one feature group at a time from the inputs, while still testing all kernel types and parameter values for every different feature list. Each time, the excluded feature group was eliminated for the GO term under consideration if the optimal performance obtained from the grid search improved with its exclusion, otherwise it was kept in. This was iterated for all feature groups. For every given kernel type, set of kernel parameter values and list of feature groups, a *k*-fold cross validation was performed using the *k* homology-based partitions of the positive and negative sets to train and test *k* classifiers. The imbalance between sizes of positive and negative sets was compensated for by setting the j parameter of the SVM-Light software to the ratio of negative to positive examples. The performance of all classifiers was measured and optimised using the Matthews Correlation Coefficient (MCC). The MCC ranges from −1 to 1, where 0 is random classification and 1 is perfect classification, and can avoid bias due to unbalanced class frequencies.
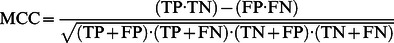
(1)


The resulting optimal values of kernel type, kernel parameters and list of feature groups were then used to train a classifier using all of the positives and negatives from the *k* partitions used in the procedure. This classifier was tested against the testing sets of proteins obtained earlier when the 30% of the positive and negative sets had been put aside. If the classifier had a value of MCC lower than 0.05, the GO term was discarded, so that such GO terms are not part of the vocabulary of FFPred 2.0. For later use, the values of sensitivity (also known as recall), specificity and precision were also calculated for the classifier.
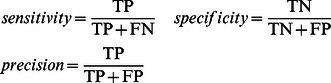
(2)


The values of all performance indicators for all classifiers are listed in Table S1.

Finally, in order to achieve optimum real world performance by using all available examples, all proteins in the GO term’s training sets (including the test proteins) were used to train a final SVM, which is the classifier actually used by FFPred 2.0 for the GO term under consideration. To do so, all example proteins were pooled together for both the positive and negative sets, and then partitioned into *k* groups as before; the best kernel type and feature group list obtained earlier were used, and the kernel parameters were optimised again with a further grid search using *k* classifiers. Using the optimal values of the kernel parameters, the final classifier for the GO term was trained on the whole pool of positive and negative examples. Note that the performance of this classifier cannot be measured exactly, but is estimated using the performance of the classifier tested earlier.

In order to estimate the posterior probability of a prediction being correct at runtime, a recent improved implementation of the classic method by Platt was used [20], [21]. Briefly, a sigmoid function was fitted to the distribution of SVM output values obtained for an appropriate set of example proteins, and estimates of the A and B parameters of the sigmoid were recorded. Such values can then be used when running FFPred 2.0 on query sequences using Eq. (3), where *y* indicates the class, and 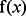 is the output of the SVM when *x* is the input.
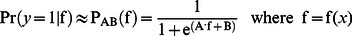
(3)


To avoid bias in the estimation, the values used for the fit were those obtained from all proteins during the final *k*-fold cross validation on the *k* classifiers with the optimal parameter set, in the last step of the training procedure [21].

Lastly, this version of FFPred was implemented so that at runtime it labels each GO term with a “higher” level of reliability if the corresponding classifier passes a further test, or with a “lower” reliability level otherwise. The test consists in having values of MCC, sensitivity, specificity, and precision higher than 0.3, 0.3, 0.7, and 0.3 respectively; these values were chosen empirically.

### Evaluation of FFPred 2.0 and Benchmarking

Benchmarking, application to model organisms and comparison to original method. Reference annotations for these analyses were obtained from UniProtKB/Swiss-Prot “complete” or “taxonomic divisions” files, release 2012_11; the same versions of CD-HIT and BLAST indicated elsewhere were used.

For all benchmarking analysis and for the comparison to the first release of FFPred, the Swiss-Prot file for human was used, and the test set of proteins was created extracting only proteins that had at least one recent “direct assay” experimental annotation (IDA evidence code) that was not present in the GOA file used for the training of FFPred 2.0. Redundancy was reduced using CD-HIT with a sequence identity threshold of 40%, resulting in a test set of 148 proteins.

Furthermore, only for the benchmarking against BLAST and ProtFun, annotations were discarded if the annotated protein or any of its homologues had been used to train the FFPred 2.0 SVM for the annotated GO term or for any of its ancestor or descendant terms. For the purpose of this analysis, proteins were considered homologues of a target if they were individuated by a BLAST search with E-value threshold equal to 10^−3^.

FFPred 1.0 predictions were obtained using the old web interface; obsolete GO terms were mapped into new ones when a single replacement term was provided via the “alternative id” or “replaced by” fields of the GO OBO file, or discarded otherwise. ProtFun predictions were obtained via the web interface at http://www.cbs.dtu.dk/services/ProtFun/; only the GO term corresponding to the category indicated by an arrow in the ProtFun output was used as a prediction for each protein. BLAST predictions were obtained transferring annotations from the best BLAST hit on a database obtained filtering the “complete” Swiss-Prot database using CD-HIT with a sequence identity threshold of 40%, and extracting only experimental annotations (EXP, IDA, IPI, IMP, IGI, IEP evidence codes).The best hit was the protein containing at least one experimental annotation and with the lowest E-value higher than 10^−3^ and 0.1 respectively for BLAST E-3 and BLAST E-1 predictors.

In the analysis on eukaryotic model organisms, for each organism (including human) the set of all proteins annotated in the corresponding Swiss-Prot “taxonomic divisions” file was filtered using CD-HIT with a sequence identity threshold of 40%, and then only proteins having at least one annotation belonging to the MF domain and one belonging to the BP domain were included in the test set.

Evaluation measures of prediction accuracy. Prediction accuracy was gauged against reference experimental annotations using two independent approaches. We carried out precision-recall analysis similar to the official assessment of the predictions submitted to the first Critical Assessment of protein Function Annotation (CAFA) experiment [22]. For a given protein s, ancestral nodes linked by “is a” relationships were added to the sets of predicted terms P_s_ and validated annotations A_s_ excluding root terms – though the assessors at CAFA made also use of other relationships between terms. Ancestors in P_s_ were assigned the maximum confidence value among those of their descendants. At a particular confidence threshold v, we then calculated: i) how many elements in P_s_ were scored at least v and were also found in A_s_ (TP_s,v_); ii) how many of them had a score higher than or equal to v but did not occur in A_s_ (FP_s,v_); iii) how many terms in A_s_ did not occur in P_s_ with a score higher than or equal to v (FN_s,v_); iv) the precision 
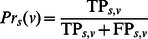
; and v) the recall 
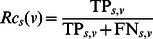
. At a given confidence threshold v, we counted the number n_v_ of proteins in the dataset D with one or more GO term assignments scored at least v, and we calculated the overall precision as 
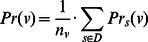
. The aggregated recall value was computed as 
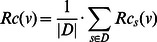
, where 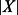 represents the number of elements in the set X. Finally, the F-measure for that confidence threshold was calculated as 
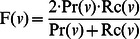
, and that was maximised over all thresholds to obtain 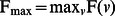.

For the comparison of FFPred 2.0 to the BLAST-based tools, alternative precision-recall plots were also obtained. The overall procedure was the same as above, however for each protein *s* the sets *P_s_* and *A_s_* contained predicted terms and validated annotations only, with no inclusion of ancestors. Then, for each confidence threshold *v*, TP*_s,v_* was calculated as the number of elements in *P_s_* that were scored at least *v* and also were found, or had an ancestor or descendant annotation, in *A_s_*; FP*_s,v_* was calculated as 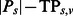, and FN*_s,v_* was calculated as 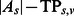.

Candidate and reference annotations were also compared through the COmbined simGIC (COGIC) scores, which have been recently proposed to take explicitly into account GO term specificity and predicted confidence values [23]. To this end, we developed a software tool (available for download at https://github.com/damianopiovesan/cogic) that initially estimates the specificity of a GO term *x* by its information content 
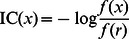
, where *r* is the root of the ontology and 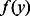 is the frequency of *y* and its descendants in the database cross-references to GO inside UniProtKB/SwissProt entries. In order to minimize assessment bias towards homology-based annotations, only GO term assignments with evidence code IEP, IPI, IMP, IGC, IGI or IDA were used [24].

The tool then compares predicted *P* and annotated *A* GO terms for a protein by defining four subsets *P_1_*, *P_2_*, *P_3_* and *P_4_* of *P* based on confidence scores greater than or equal to 0.75, 0.50, 0.25 and 0, respectively. All ancestral nodes are added to such sets as well as to the *A*, and the simGIC scores [25] *S*_1_, *S*_2_, *S*_3_, and *S*_4_ are computed from separate comparison of *A* with *P*_1_, *P*_2_, *P*_3_ and *P*_4_ as
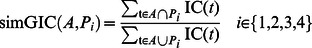
(4)


The output COGIC score is
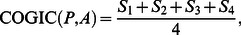
(5)and takes a value in the range [0,1], giving higher credit to correct predictions with higher confidence scores. The COGIC score is 1 if and only if all predicted GO terms are validated and their confidence scores are greater than or equal to 0.75. It equals 0 if and only if the root of the ontology is the only element shared by *P* and *A*.

## Results and Discussion

### An Extended Vocabulary of Binding Activities and Biological Processes

FFPred 2.0 makes feature-based assignments of molecular function (MF) and biological process (BP) categories by exploiting updated public resources and tools. The impressive growth of protein sequence and annotation databases has allowed us to apply more restrictive conditions, particularly aimed at the confident selection of negative examples for training. Additionally, some feature predictors have been updated to the latest release, which is expected to further enhance functional inference accuracy.

**Figure 1:**
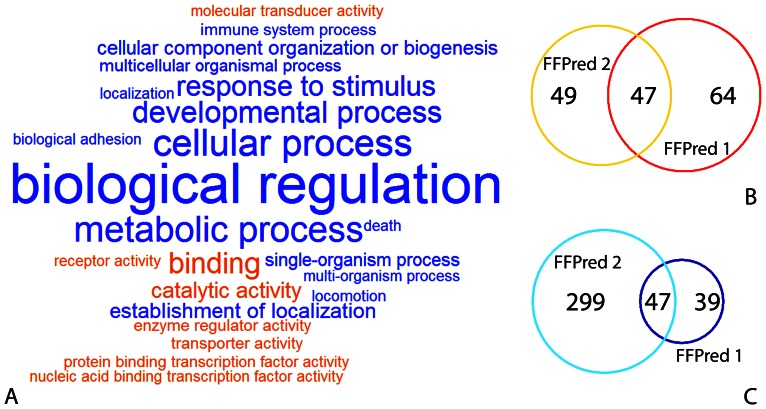
Make up of FFPred 2.0 GO term list and differences from the previous release. High-level summary of the FFPred 2.0 GO term vocabulary: each tag in the cloud is a child of the root of the MF or BP domains (shown in orange and blue respectively), and their size reflects how many of their descendants can be predicted (A). Extent of the overlap between FFPred 1.0 and FFPred 2.0 GO term lists in the MF (B) and BP (C) domains.

Figure 1 summarizes the key features of the new vocabulary and the changes from the previous one. The updated SVM library provides models for 442 GO terms and largely extends the coverage of the ontology – and of the BP domain in particular – by focusing on molecular recognition and its role in regulatory, signalling, developmental and metabolic processes. Compared to the first release, the GO term dictionary has considerably grown in size, but not through a straightforward extension of the earlier version: the number of MF categories has dropped from 111 to 96 and, indeed, the new list shares only 94 items out of the original 197. This stems partly from the revised training procedures, and partly from the changes in GO term definitions and in the ontology organization, which follow the accumulation of new biological knowledge over time. Finally, despite the more stringent criteria for GO term selection, the new list does not differ substantially in specificity from that previously used (see Figure S1).

As of December 2012, UniProtKB/Swiss-Prot curators have discontinued using some GO terms in their annotations, including 20 MF terms that either FFPred version can assign to input amino acid sequences. Such terms are not considered in the following analyses, although they are still part of the online server output.

### Performance Evaluation and Improvement Over the Original FFPred

The performance of the SVM corresponding to each GO term was measured using the MCC and other performance indicators, as reported in Table S1. Values of MCC for SVMs range between 0.052 and 0.956 with an average of 0.375, and there are several high-performing GO terms, with 28 of them (19 MF terms, 9 BP terms) having MCC values higher than 0.7. GO terms in the vocabulary of FFPred 2.0 are sorted into two different categories with higher and lower level of reliability, according to whether or not the corresponding SVM had high values of MCC, sensitivity, specificity, and precision. This distinction is clearly indicated in the web server results page, where results concerning GO terms with a lower level of reliability are displayed against a red background. Overall, there are 200 GO terms in the “higher reliability” set (72 in the MF domain, 128 in the BP domain), and the corresponding SVMs have an average MCC of 0.538; the remaining 242 GO terms are in the other set (24 MF, 218 BP), with an average MCC of 0.240. It is evident that GO terms in the MF domain tend to be more reliably predicted – although they are less numerous than the BP terms.

The performances of SVMs for 39 GO terms that are common to the vocabularies of FFPred 1.0 and FFPred 2.0, and for which the FFPred 1.0 data is available, are compared in Table 2. With the exception of three cases, these GO terms can be predicted more confidently now than before, with an average 40% increase in MCC value.

**Table 2:** Comparison of MCC values for common GO terms.

GO term	Description	Domain	MCC – FFPred 1.0	MCC – FFPred 2.0
GO:0000166	nucleotide binding	MF	0.361	0.63
GO:0003677	DNA binding	MF	0.568	0.622
GO:0003700	sequence-specific DNA binding transcription factor activity	MF	0.538	0.741
GO:0004252	serine-type endopeptidase activity	MF	0.719	0.732
GO:0004672	protein kinase activity	MF	0.429	0.616
GO:0004674	protein serine/threonine kinase activity	MF	0.479	0.684
GO:0004713	protein tyrosine kinase activity	MF	0.488	0.666
GO:0004866	endopeptidase inhibitor activity	MF	0.568	0.576
GO:0004871	signal transducer activity	MF	0.646	0.719
GO:0004872	receptor activity	MF	0.429	0.751
GO:0004888	transmembrane signaling receptor activity	MF	0.526	0.809
GO:0004930	G-protein coupled receptor activity	MF	0.706	0.896
GO:0005125	cytokine activity	MF	0.558	0.711
GO:0005215	transporter activity	MF	0.390	0.748
GO:0005261	cation channel activity	MF	0.447	0.624
GO:0008083	growth factor activity	MF	0.346	0.568
GO:0008233	peptidase activity	MF	0.309	0.531
GO:0008236	serine-type peptidase activity	MF	0.711	0.721
GO:0016773	phosphotransferase activity, alcohol group as acceptor	MF	0.339	0.607
GO:0006351	transcription, DNA-dependent	BP	0.566	0.645
GO:0006355	regulation of transcription, DNA-dependent	BP	0.581	0.517
GO:0006468	protein phosphorylation	BP	0.372	0.574
GO:0006486	protein glycosylation	BP	0.414	0.661
GO:0006796	phosphate-containing compound metabolic process	BP	0.348	0.516
GO:0006810	transport	BP	0.306	0.509
GO:0006811	ion transport	BP	0.370	0.738
GO:0006812	cation transport	BP	0.315	0.687
GO:0007155	cell adhesion	BP	0.371	0.610
GO:0007156	homophilic cell adhesion	BP	0.714	0.916
GO:0007165	signal transduction	BP	0.319	0.211
GO:0007166	cell surface receptor signaling pathway	BP	0.525	0.448
GO:0007169	transmembrane receptor protein tyrosine kinase signaling pathway	BP	0.343	0.484
GO:0007186	G-protein coupled receptor signaling pathway	BP	0.724	0.817
GO:0007606	sensory perception of chemical stimulus	BP	0.685	0.942
GO:0007608	sensory perception of smell	BP	0.730	0.956
GO:0009101	glycoprotein biosynthetic process	BP	0.417	0.641
GO:0016310	phosphorylation	BP	0.321	0.496
GO:0016337	cell-cell adhesion	BP	0.578	0.636
GO:0016567	protein ubiquitination	BP	0.303	0.417

For each GO term, the table reports its GO domain, its description, and the MCC values of its corresponding SVMs within FFPred 1.0 and FFPred 2.0.

Comparison to original FFPred on a common set of recently annotated human proteins. A direct comparison on a common set of target proteins shows that FFPred 2.0 performs better than FFPred 1.0. Both tools were run on a set of 148 human proteins that were annotated on the UniProtKB/Swiss-Prot database with experimental annotations after the training of FFPred 2.0. In order to avoid bias in favour of one tool or the other, all annotations were used in the analysis, includes annotations used for the training of either tool: this allows a realistic evaluation of the output that a putative user would obtain from the two servers.

**Figure 2:**
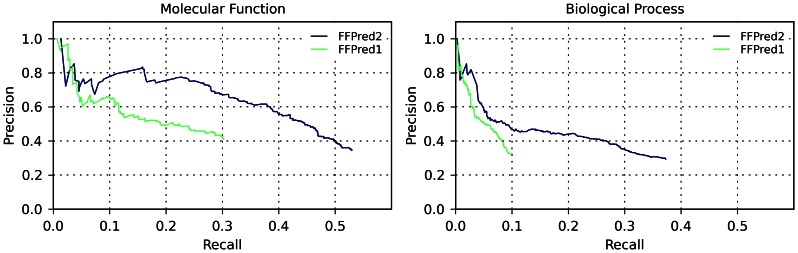
Comparison of FFPred 1.0 and FFPred 2.0 by means of precision-recall plots. Precision-recall plots comparing the performance of FFPred 1.0 and FFPred 2.0 on a common dataset of 148 human proteins, for predictions in the MF domain (left) and BP domain (right).

The predictions were evaluated using precision-recall plots (Figure 2) and by means of COGIC scores (Figure S2). For both domains, the precision of FFPred 2.0 predictions is usually higher than that of FFPred 1.0 predictions at the same level of recall, and the range spanned in terms of recall is much wider for the new version of FFPred. Moreover, for the MF domain, the median COGIC score increases more than 3-fold from FFPred 1.0 to FFPred 2.0 (0.061 to 0.225), while for the BP domain it increases more than 6-fold (0.018 to 0.113), denoting a marked improvement from the old to the new version of FFPred.

These results prove the benefit of retraining and updating FFPred on a regular basis, as databases of functional annotations change and increase in size, and updated or more sophisticated tools for predicting features are released.

### Benchmarking on Human Proteins

A complete benchmark test was performed using the new version of FFPred, two BLAST-based tools, and the ProtFun server [9], which is the other main publicly available tool for protein function prediction based on sequence features. The test set of proteins was the same used for the comparison between FFPred 1.0 and FFPred 2.0 above. However, all annotations to targets that were homologue to proteins used in the training procedure were excluded from the analysis, in order to replicate the situation where no homology information is available to users of FFPred 2.0. The BLAST tools were able to transfer annotations from database proteins with E-value higher than 10^−3^ (“BLAST E-3” tool) or 0.1 (“BLAST E-1” tool), mimicking two situations with little homology information left.

The benchmark test using the two BLAST tools and the comparison to ProtFun were kept separate. The first test involved tools of a different nature, as the BLAST-based tools make use of direct albeit limited homology information to reassign pre-existing annotations, while FFPred 2.0 does not require any annotations to be present in the underlying protein database. In fact, the test was useful to point out how much of the performance of tools performing homology-based transfer FFPred 2.0 can achieve. In contrast, the second test was a direct comparison between ProtFun and FFPred 2.0. For ProtFun, both the “Gene Ontology category” and “Enzyme” predictions were kept into account.

#### Comparison to BLAST-based tools

**Figure 3:**
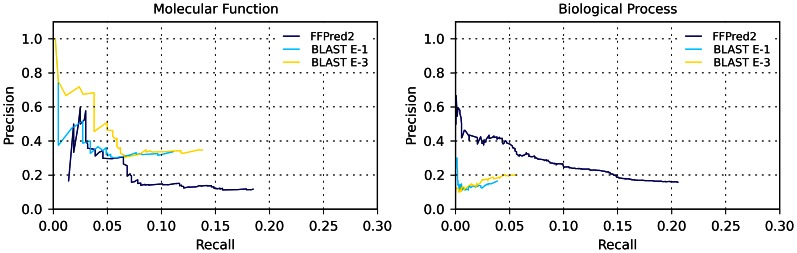
Comparison of FFPred 2.0 and BLAST-based tools by means of precision-recall plots. Precision-recall plots comparing the performance of FFPred 2.0 to the BLAST-based tools described in the main text on a common dataset of 148 human proteins, for predictions in the MF domain (left) and BP domain (right).

The outcome of the test against the BLAST tools was analysed using precision-recall plots, as depicted in Figure 3. For the MF domain, the maximum F-measure is equal to 0.144 for FFPred 2.0, while it evaluates to 0.167 and 0.197 for the E-1 and E-3 BLAST tools respectively; similarly, the overall curves suggest that FFPred 2.0 can recover most of the precision of homology-based tools in these predictions. For the BP domain both the overall precision-recall curve and the values of F_max_ (equal to 0.180, 0.087, 0.063 respectively for FFPred 2.0, BLAST E-3, BLAST E-1) actually denote a much higher accuracy for FFPred 2.0 predictions compared to those obtained with BLAST-based tools. Moreover, for a complementary comparison, alternative precision-recall plots are shown in Figure S3. These employ a very simple measure of the accuracy of predictions: a prediction is considered correct if and only if the predicted GO term is an ancestor term (a correct, but generic prediction) or a descendant term (a compatible prediction) of a reference annotation for the target protein. This compensates for the fact that most GO terms in the FFPred 2.0 vocabulary are far more generic than the average annotations transferred by BLAST, which gave FFPred 2.0 an evident disadvantage in the standard evaluation. Using this measure, the performance of FFPred 2.0 overwhelms the one of BLAST-based tools for both GO domains. These results suggest that often the relatively poorer performance obtained in the standard analysis of MF predictions was due to FFPred 2.0 not being able to predict terms that were deep enough in the GO graph, although the high-level predictions were correct.

Finally, the COGIC score plots for this benchmark test are provided in Figure S4 for both GO domains. The analysis highlights a comparable performance for the MF domain, while for the BP domain FFPred 2.0 achieves higher scores, with an increase of more than 400% in the median value to the BLAST E-3 and E-1 tools.

This analysis shows the usefulness of FFPred 2.0 predictions, which are independent of the annotations present in the database when a query sequence is processed. In practice, for effective functional annotation in the BP domain FFPred 2.0 can be used as the superior method. For MF annotation, users will usually exploit homology information available on the query sequence first; however, especially when little or no information is available, FFPred 2.0 is shown to be very valuable in complementing such knowledge with novel predictions of functional annotation.

Comparison to ProtFun. In the case of the direct comparison to ProtFun on the same dataset, the precision-recall plots (shown in Figure S5) describe a better performance of FFPred 2.0. For predictions in the MF domain, both ranges of precision and recall are better for FFPred 2.0, and often the precision values are higher at the same value of recall; the improvement is more striking for the BP domain. This is exemplified by the maximum F-measure values: for predictions in the MF domain, F_max_ is equal to 0.144 and 0.049 respectively for FFPred 2.0 and ProtFun, while for predictions in the BP domain F_max_ is equal to 0.180 and 0.008 respectively. Moreover, COGIC scores box plots (reported in Figure S6) show how the median score achieved on this dataset was much higher for FFPred 2.0 compared to ProtFun, for both GO domains. In summary, both measures of prediction accuracy highlight an overall higher performance of FFPred 2.0 in this case.

### Use of FFPred 2.0 with Other Eukaryotic Organisms

**Figure 4:**
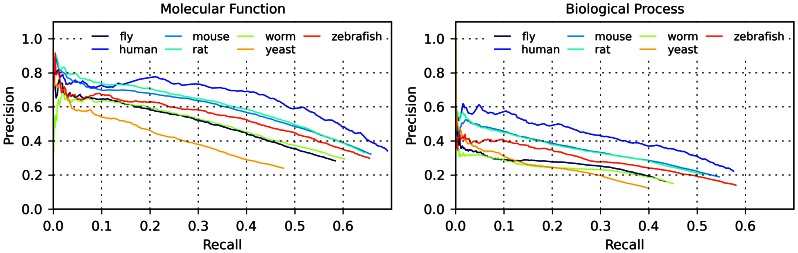
Evaluation of FFPred 2.0 on eukaryotic proteomes by means of precision-recall plots. Precision-recall plots describing the performance of FFPred 2.0 on several eukaryotic proteomes, for predictions in the MF domain (left) and BP domain (right).

FFPred 2.0 was trained using human data. However, in order to more generally assess its usefulness in predicting functional annotations, this version of FFPred was tested on several other eukaryotic model organisms: mouse (*Mus musculus*), rat (*Rattus norvegicus*), fly (*Drosophila melanogaster*), zebrafish (*Danio rerio*), worm (*Caenorhabditis elegans*), and yeast (*Saccharomyces cerevisiae*). For each of these organisms plus human, we evaluated the performance of FFPred 2.0 on a dataset containing all proteins with annotations both in the MF and in the BP domains taken from a non-redundant version of the whole proteome. The results of the analysis are shown in Figure 4 (precision-recall plots) and Figure S7 (plots of COGIC scores).

The performance of FFPred 2.0 on all model organisms is comparable to that achieved on human targets, and therefore FFPred 2.0 can be effectively considered a useful tool for general eukaryotic function prediction. The classification accuracy decreases for species with higher evolutionary distance to human; this can be expected for example due to decreasing of conservation of some of the analysed sequence features between proteins from distant eukaryotic proteomes, and to variable comprehensiveness of annotation databases for different organisms. However, the precision-recall plots show that the extent of this decrease is just 11% (precision at 30% recall, for rat) to 48% (for yeast) compared to human for MF predictions, and 23% (for mouse) to 54% (for yeast) for BP predictions. Similarly, the median COGIC score only decreases by 12% (for mouse) to 63% (for yeast) of the value for human in the case of MF predictions, and by 26% (for mouse) to 56% (for yeast) in the case of BP predictions.

### Visualisation of the Results

**Figure 5:**
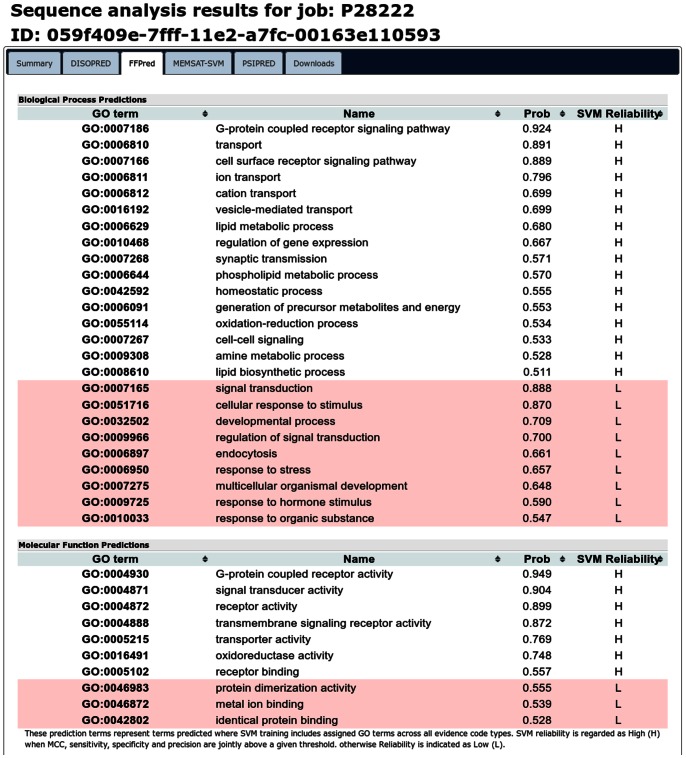
Sample FFPred 2.0 output within the PSIPRED web server. Top part of the FFPred 2.0 tab within the results page for the submission of the sequence of Serotonin receptor 1B to the PSIPRED Protein Analysis Workbench web server. Predicted GO term assignments are listed together with the associated posterior probability and SVM reliability level.

FFPred 2.0 runs within the web servers of the UCL Bioinformatics Group [26]; users can access the interface of the PSIPRED Protein Analysis Workbench at http://bioinf.cs.ucl.ac.uk/psipred/and select the “FFPred v2.0” option. Figure 5 shows the content of the top half of the “FFPred” tab within the results page for a sample protein, Serotonin receptor 1B (UniProtKB primary accession number: P28222). For each of the two GO domains under consideration, a table lists all terms that were predicted for the query sequence: each line shows the GO term code and description, the posterior probability associated to the prediction itself, and the intrinsic reliability level of the SVM corresponding to that GO term (see Materials and Methods, end of section “Training of the SVMs”). In the default view of the “FFPred” results tab, predictions of GO terms from higher reliability SVM models (“H”) are listed first, while predictions of GO terms from lower reliability models (“L”) are listed below against a red background, and can be interpreted as further, relatively less safe suggestions for functional characterisation. Predictions can however be sorted according to the values of any column.

It is worth noting that the intrinsic reliability level associated with a given GO term SVM is independent of the query sequence, since it depends on the quality of the SVM model only. The actual confidence level of each individual prediction for the query protein is instead indicated by the posterior probability value. Compared to the output of the previous version of FFPred, the interpretation of the results is more straightforward, due to the absence of a “jury” value. For FFPred 1.0 predictions, such value described the consensus of several SVMs that were run for the prediction of an individual GO term. This is not needed any more, since for each GO term only a single SVM is used. The SVM output is converted into the posterior probability that the query protein is actually annotated with that GO term. The corresponding annotation is therefore displayed whenever such probability is higher than 0.5.

The full content of the “FFPred” results tab is reported in Figure S8. The rest of the web page has not been modified for this version of FFPred: it displays more detailed information about the feature content of the query sequence, that was used for the GO term assignments. Moreover, users can now use the “Download” results tab to obtain copies of the list of predictions and the list of all posterior probabilities corresponding to the 442 GO terms, both in plain text format.

Finally, a standalone version of FFPred 2.0 can be downloaded from the group’s download webpages (http://bioinfadmin.cs.ucl.ac.uk/downloads/ffpred/). As FFPred 2.0 relies on several pieces of third party software, detailed instructions and suggestions for configuring the standalone version can be found in the accompanying documentation.

## Supporting Information

Figure S1 **Specificity distribution of the vocabulary terms of FFPred 1.0 and FFPred 2.0.** For the old and new versions of FFPred, the histogram shows the vocabulary specificity, which is measured for each term as the minimum distance from the root of the Ontology. Both MF and BP terms are considered together in each distribution.
(TIF)

Figure S2 **Comparison of FFPred 1.0 and FFPred 2.0 by means of COGIC scores.** Box plots depicting the distribution of values of COGIC scores on a common test set of 148 human proteins for FFPred 1.0 and FFPred 2.0, for predictions in the MF domain (left) and BP domain (right).
(TIF)

Figure S3 **Alternative comparison of FFPred 2.0 and BLAST-based tools by means of precision-recall plots.** Precision-recall plots comparing the performance of FFPred 2.0 to the BLAST-based tools on a common dataset of 148 human proteins, for predictions in the MF domain (left) and BP domain (right). An alternative measure is used here, that simply considers each prediction correct if and only if it assigns a GO term that is an ancestor term or a descendant term of a reference annotation of the target protein.
(TIF)

Figure S4 **Comparison of FFPred 2.0 and BLAST-based tools by means of COGIC scores.** Box plots depicting the distribution of values of COGIC scores on a common test set of 148 human proteins for FFPred 2.0 and the BLAST-based tools described in the main text, for predictions in the MF domain (left) and BP domain (right).
(TIF)

Figure S5 **Comparison of FFPred 2.0 and ProtFun by means of precision-recall plots.** Precision-recall plots comparing the performances of FFPred 2.0 and ProtFun on a common dataset of 148 human proteins, for predictions in the MF domain (left) and BP domain (right).
(TIF)

Figure S6 **Comparison of FFPred 2.0 and ProtFun by means of COGIC scores.** Box plots depicting the distribution of values of COGIC scores on a common test set of 148 human proteins for FFPred 2.0 and ProtFun, for predictions in the MF domain (left) and BP domain (right).
(TIF)

Figure S7 **Evaluation of FFPred 2.0 on eukaryotic proteomes by means of COGIC scores.** Box plots depicting the distribution of values of COGIC scores obtained by FFPred 2.0 on several eukaryotic proteomes, for predictions in the MF domain (left) and BP domain (right).
(TIF)

Figure S8 ***Sample FFPred 2.0 output page.*** Complete FFPred 2.0 output as shown in the FFPred tab within the results page for the submission of the sequence of Serotonin receptor 1B to the PSIPRED Protein Analysis Workbench web server. After the list of predicted GO term assignments, the feature content of the query sequence is shown, as in the previous version of FFPred.
(TIF)

Table S1 ***FFPred 2.0 vocabulary and SVM performance.*** The table lists all GO terms that are present in the vocabulary of FFPred 2.0, together with the values of MCC, sensitivity, specificity, and precision obtained by the corresponding SVMs during the training procedure.
(XLSX)
