# Supplementary figures and images for: FFPred 2.0: Improved Homology-Independent Prediction of Gene Ontology Terms for Eukaryotic Protein Sequences
Source: PLoS One. 2013 May 22;8(5):e63754. doi: 10.1371/journal.pone.0063754 (PMC3661659; doi:10.1371/journal.pone.0063754)

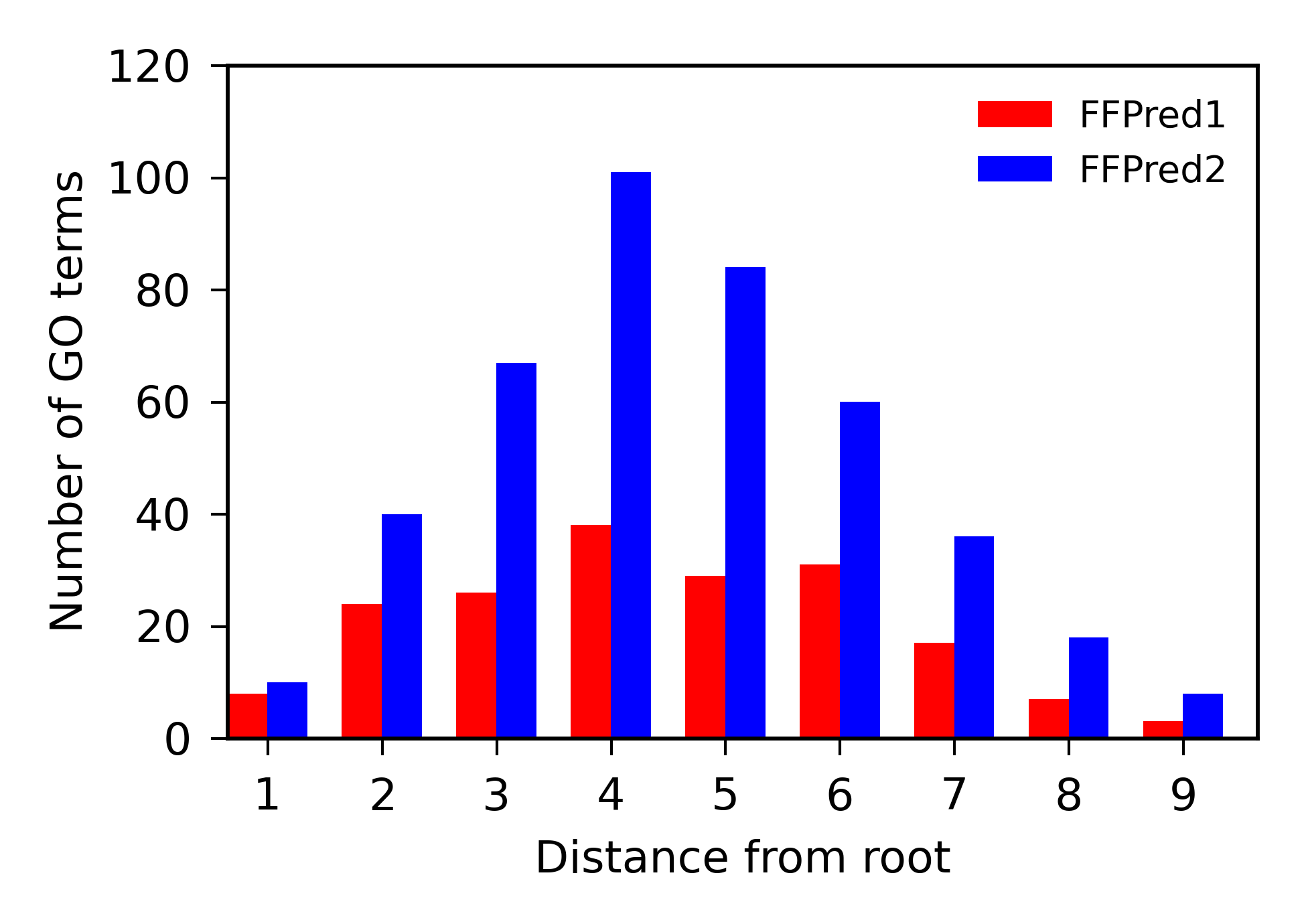

Supplement: Figure S1 — Specificity distribution of the vocabulary terms of FFPred 1.0 and FFPred 2.0. For the old and new versions of FFPred, the histogram shows the vocabulary specificity, which is measured for each term as the minimum distance from the root of the Ontology. Both MF and BP terms are considered together in each distribution. (TIF) [file pone.0063754.s001.tif]

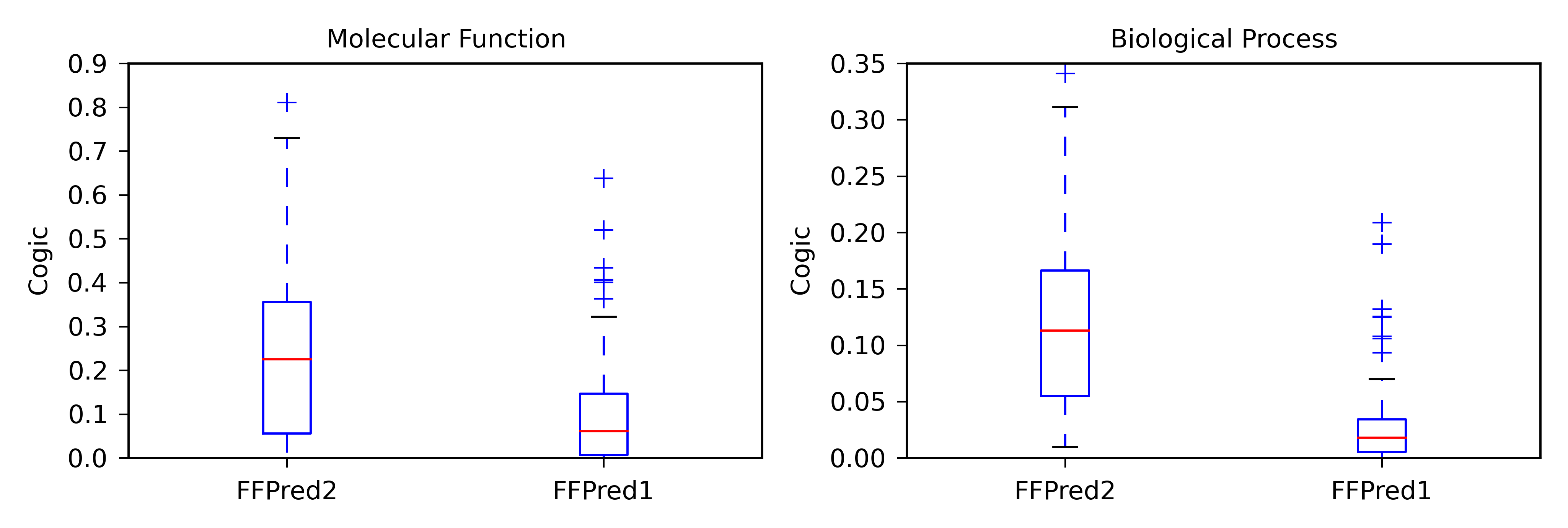

Supplement: Figure S2 — Comparison of FFPred 1.0 and FFPred 2.0 by means of COGIC scores. Box plots depicting the distribution of values of COGIC scores on a common test set of 148 human proteins for FFPred 1.0 and FFPred 2.0, for predictions in the MF domain (left) and BP domain (right). (TIF) [file pone.0063754.s002.tif]

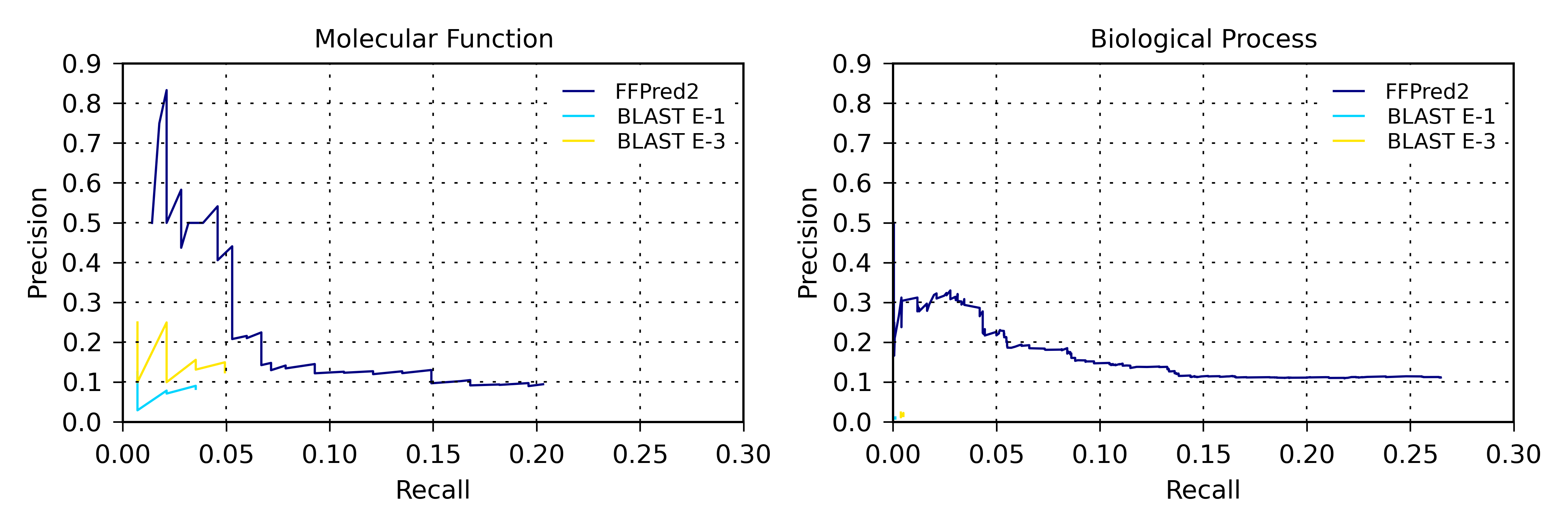

Supplement: Figure S3 — Alternative comparison of FFPred 2.0 and BLAST-based tools by means of precision-recall plots. Precision-recall plots comparing the performance of FFPred 2.0 to the BLAST-based tools on a common dataset of 148 human proteins, for predictions in the MF domain (left) and BP domain (right). An alternative measure is used here, that simply considers each prediction correct if and only if it assigns a GO term that is an ancestor term or a descendant term of a reference annotation of the target protein. (TIF) [file pone.0063754.s003.tif]

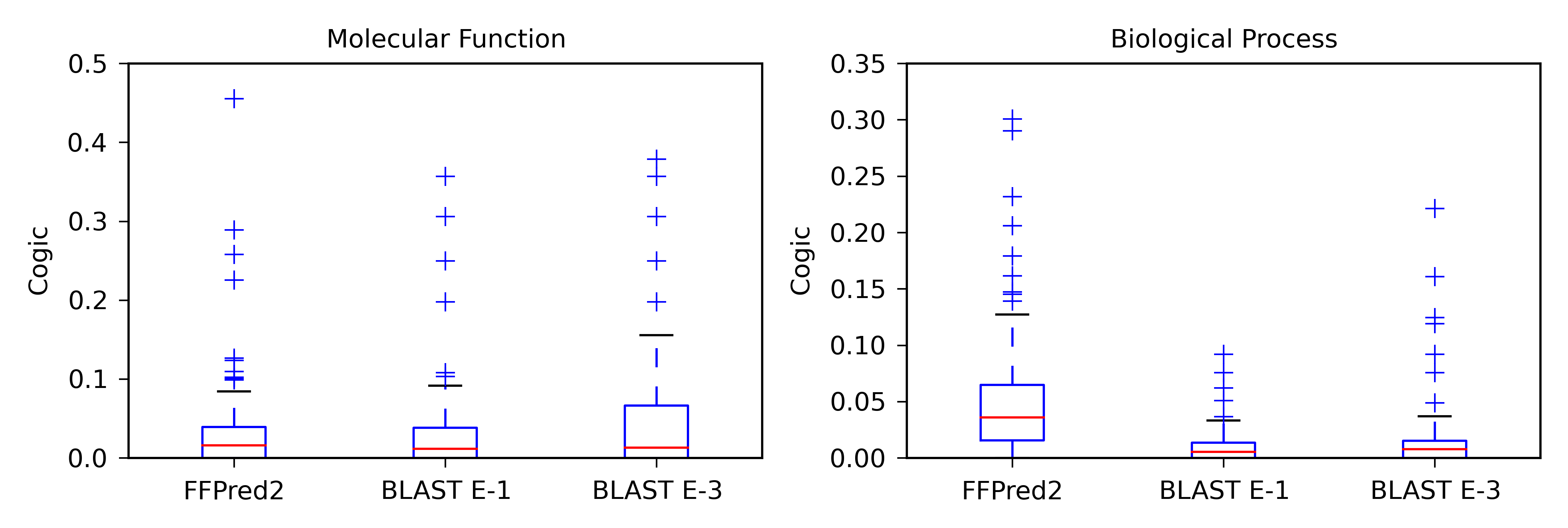

Supplement: Figure S4 — Comparison of FFPred 2.0 and BLAST-based tools by means of COGIC scores. Box plots depicting the distribution of values of COGIC scores on a common test set of 148 human proteins for FFPred 2.0 and the BLAST-based tools described in the main text, for predictions in the MF domain (left) and BP domain (right). (TIF) [file pone.0063754.s004.tif]

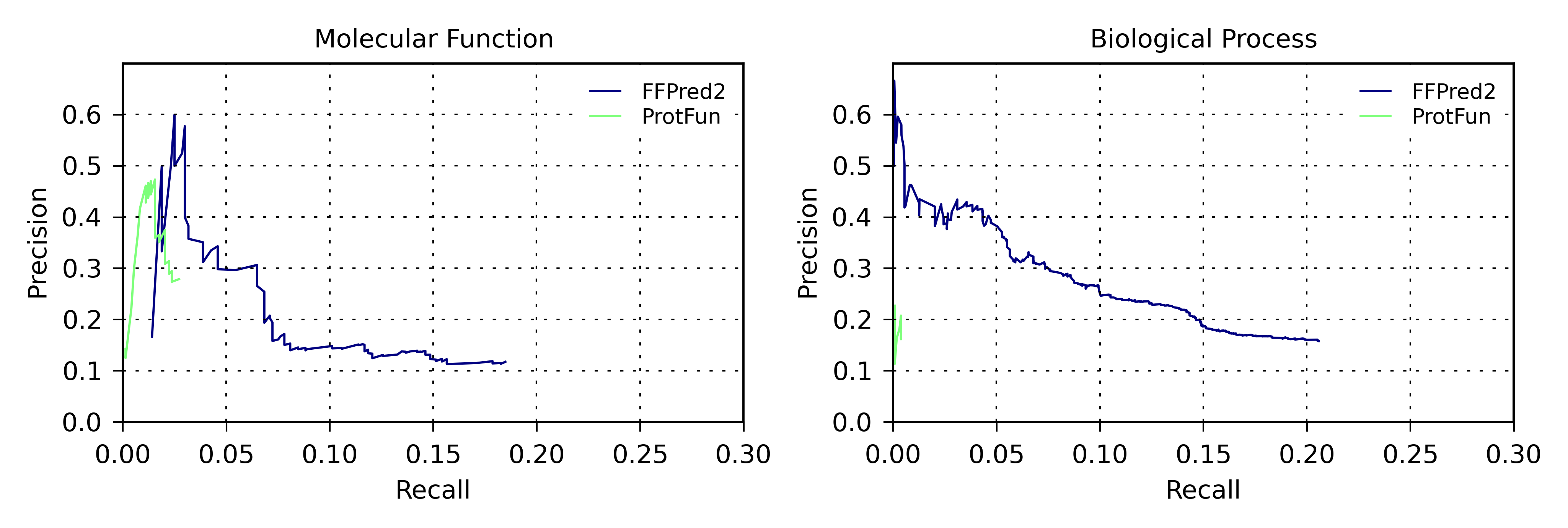

Supplement: Figure S5 — Comparison of FFPred 2.0 and ProtFun by means of precision-recall plots. Precision-recall plots comparing the performances of FFPred 2.0 and ProtFun on a common dataset of 148 human proteins, for predictions in the MF domain (left) and BP domain (right). (TIF) [file pone.0063754.s005.tif]

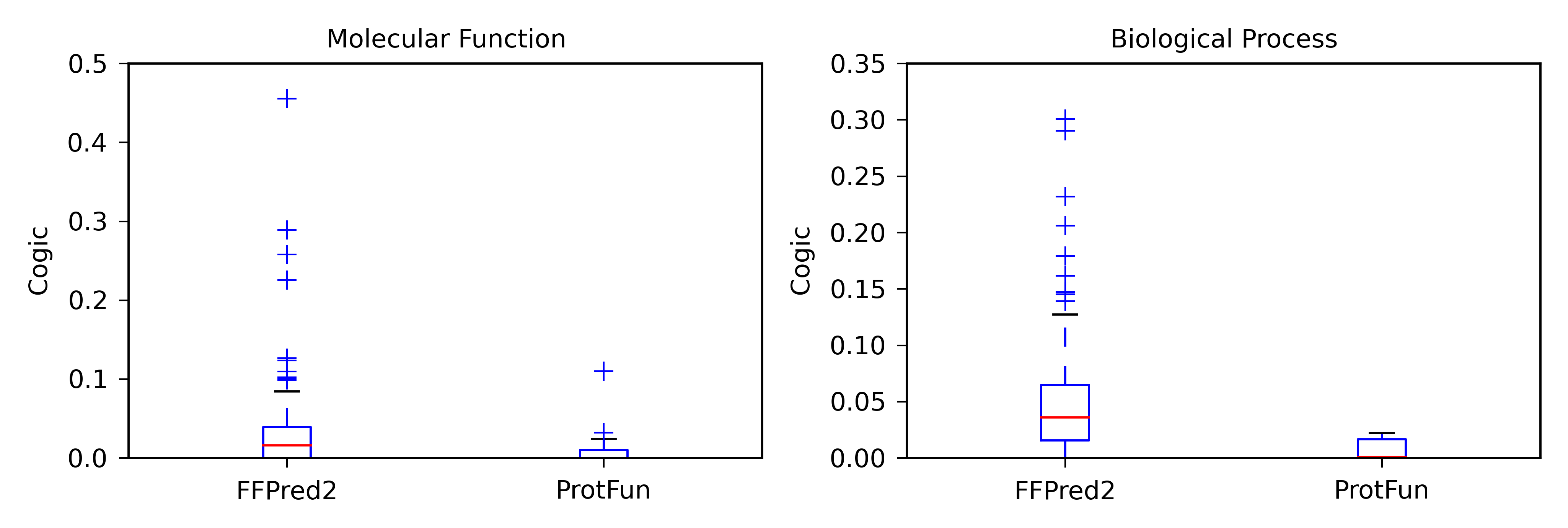

Supplement: Figure S6 — Comparison of FFPred 2.0 and ProtFun by means of COGIC scores. Box plots depicting the distribution of values of COGIC scores on a common test set of 148 human proteins for FFPred 2.0 and ProtFun, for predictions in the MF domain (left) and BP domain (right). (TIF) [file pone.0063754.s006.tif]

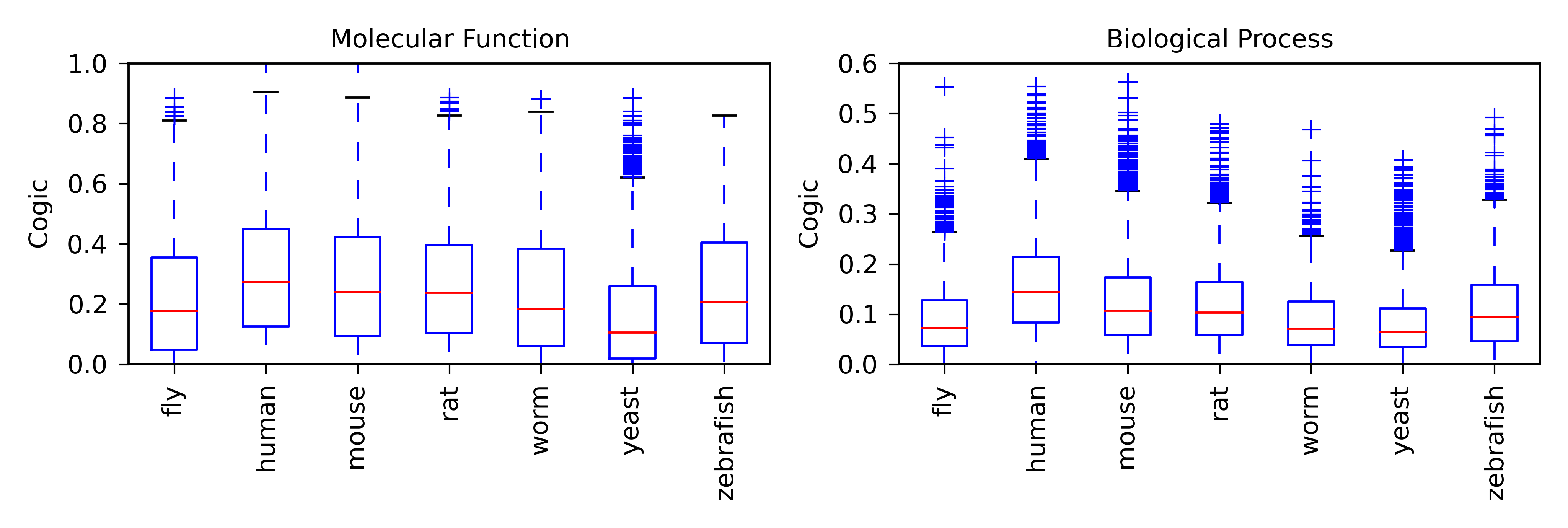

Supplement: Figure S7 — Evaluation of FFPred 2.0 on eukaryotic proteomes by means of COGIC scores. Box plots depicting the distribution of values of COGIC scores obtained by FFPred 2.0 on several eukaryotic proteomes, for predictions in the MF domain (left) and BP domain (right). (TIF) [file pone.0063754.s007.tif]

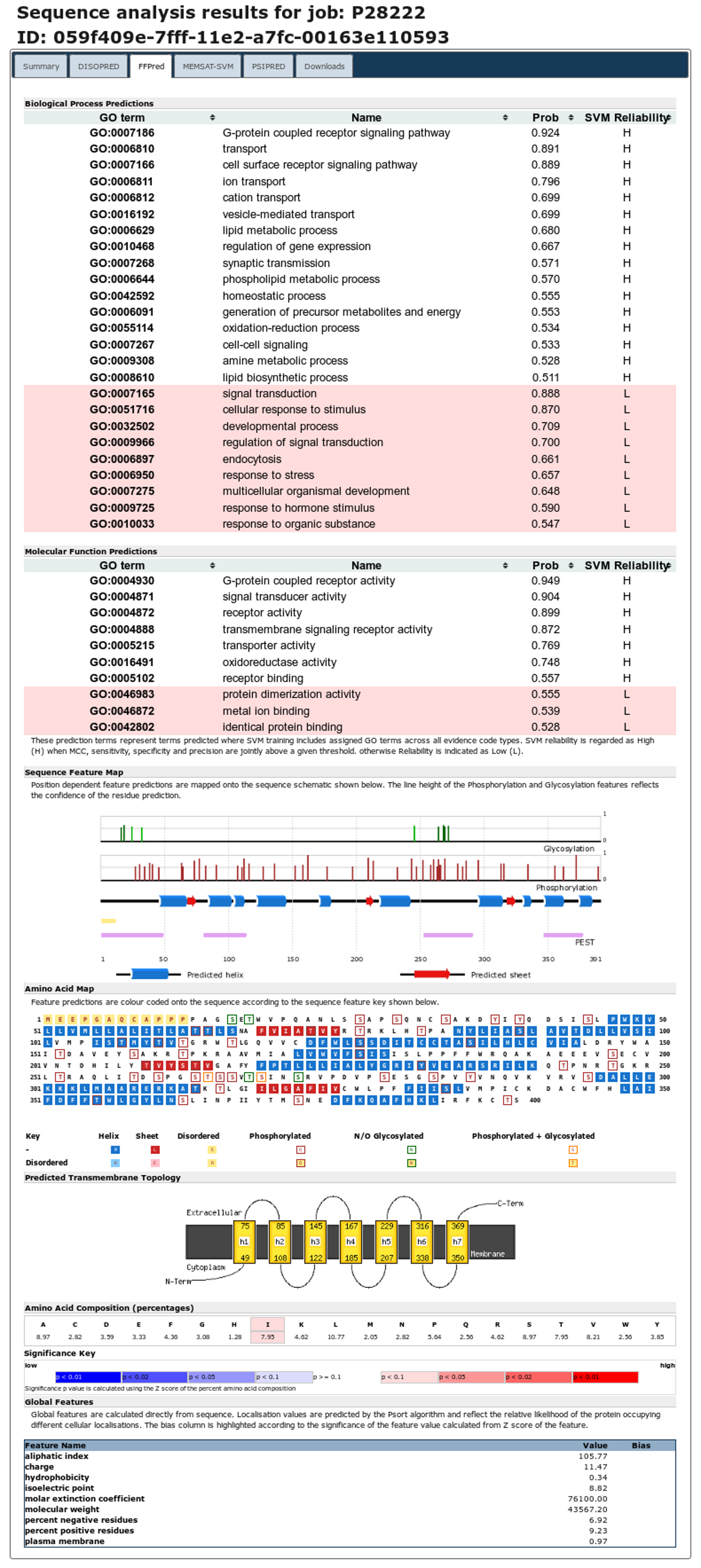

Supplement: Figure S8 — Sample FFPred 2.0 output page. Complete FFPred 2.0 output as shown in the FFPred tab within the results page for the submission of the sequence of Serotonin receptor 1B to the PSIPRED Protein Analysis Workbench web server. After the list of predicted GO term assignments, the feature content of the query sequence is shown, as in the previous version of FFPred. (TIF) [file pone.0063754.s008.tif]
